# Additive conjoint measurement and the resistance toward falsifiability in psychology

**DOI:** 10.3389/fpsyg.2013.00246

**Published:** 2013-05-06

**Authors:** Moritz Heene

**Affiliations:** Department of Psychology, Learning Sciences Research Methodology, Ludwig Maximilian University of MunichMunich, Germany

The history of the past four decades of the theory and application of additive conjoint measurement (ACM) is characterized by vivid developments of its theoretical foundation (cf. Luce and Tukey, 1964; Krantz et al., 1971, 2006; Narens, 1974), industrious developments of statistical and computational implementations (cf. Karabatsos and Ullrich, 2002; Karabatsos and Sheu, 2004; Karabatsos, 2005; Myung et al., 2005) and heated debates about its applicability and significance in psychology (cf. Michell, 1997, 2009; Borsboom and Mellenbergh, 2004; Barrett, 2008; Borsboom and Scholten, 2008; Kyngdon, 2008a; Trendler, 2009). What started as a promising foundation to solve the everlasting debate about the quantitative nature of psychological attributes (Ferguson et al., 1939) ended in perseverative debates with very little transfer to mainstream psychological science still being dominated by structural equation modeling (SEM) and item response theory (IRT). After reading the aforementioned articles, and comparing their implications with the day-to-day business of mainstream psychological science, even an unbiased reader would certainly agree with Cliff (1992) that ACM was a “… revolution that never happened” (p. 186).

It is not the aim of this article, to discredit the efforts of mathematical psychology and proponents of ACM in particular. I just want to address the naïve but relevant question why ACM as a stringent way to formalize and to test the requirements of quantitative measurement in psychology has not been embraced by mainstream psychology as a means to an end to test what they always claim: that most of the attributes (e.g., intelligence and personality factors) are quantitative.

An attribute possessing a quantitative structure is required to satisfy the three conditions of ordinality (transitivity, antisymmetry, and strong connexity) *and* the six conditions of additivity (associativity, commutativity, monotonicity, solvability, positivity, and the Archimedean condition; cf. Michell, 1990, p. 52f.). Most of these conditions are *testable* hypotheses but I have never seen any empirical test in psychological articles before data were analyzed with SEM or IRT models, which already *assume* the quantitative structure of the attributes under consideration as argued below. Somewhere during my psychology studies at the university I learned that psychology is an empirical science and that there is therefore no room for claims that should just be believed. However, given the assumed but almost never tested quantitative nature of most of the psychological attributes as reflected in factor analysis, SEM and IRT models, I must have missed or misunderstood something.

## Resistance toward inconvenient truth

The question arises why debates about testing the assumption of quantitative measurement more rigorously emerge from time to time without any broader impact on psychological measurement with a few exceptions (Luce, 2000; Kyngdon, 2011). Any attempt to answer this question will, of course, be incomplete, so that I will suggest a factor that might be of special importance: psychologist's avoidance toward falsifiability and hence, toward inconvenient truth.

A number of authors state (cf. Borsboom and Mellenbergh, 2004; Borsboom and Scholten, 2008; Fisher, 2011) that the axiomatic structure of ACM is too restrictive with respect to the regularities in the order relations of the items, the examinees, and an ordinal index of the probability of a correct response. ACM relates to situations in which one attribute (*P*; e.g., the probability of getting an item correct) is related *additively* to two others (*A* the ability and *B* the item difficulty) such that *P* = *f*(*A* + *B*) (where *f* is any positive monotonic function). In fact, the requirements of ACM are rarely fulfilled in applied psychological data (Cliff, 1992; Michell, 2009) because the data must satisfy the highly restrictive conditions of double cancelation, solvability, and the Archimedian axiom (cf. Michell, 1990). Satisfaction of these requirements implies that *A* and *B* are additive and are therefore quantitative (cf. Krantz et al., 1971).

I therefore agree with the argument that it is more than questionable why such rigorous measurement structures could be found in psychological data. As illustrated elsewhere (cf. Schönemann, 1994; Heene, 2011) psychology seemed to be overwhelmed by the successful application of mathematics in classical physics and invented “… models with close reference to those of classical physics, which were *then* applied to psychological observations” (Heene, 2011, p. 53; italics in the original). This approach ignores that the development of mathematical models has been closely interwoven with the empirical observation of invariant phenomena in physics implying that the mathematical models have often been derived *from* those phenomena (see also Sherry, 2011).

On the other hand, the tools of mainstream psychology such as SEM and IRT make exactly these strong assumptions about the quantitative structure of psychological attributes. But avoiding any tests of quantitative measurement but applying methods making the assumption of quantity appears to be nothing more than a self-delusion that one bears something valuable instead of being in fact empty-handed. This all too strong tendency to avoid falsification is probably deeply rooted in the scientifically unhealthy political/economical aspiration of psychology (Vautier et al., 2012) which keeps the machine for paper-producing and grant-funding well-oiled but also leading to a severe publication bias. Consider Levine et al. (2009) who showed that effect size and sample size are negatively correlated in 80% of meta-analyses. Consider Fanelli (2010, p. 4) who found that “… the odds of reporting a positive result were around five times higher for papers published in Psychology and Psychiatry and Economics and Business than in Space Science” (see also Fanelli, 2009, 2012; Bones, 2012). Despite these numbers, the possibly best evidence of my claims comes from a logical argument: has anyone ever seen articles using SEM, IRT, or Rasch models in which the author admitted the *falsification* of his/her hypotheses? On the contrary, it appears that stringent model tests are mostly carefully avoided in favor of insensitive “goodness-of-fit indices” (cf. Karabatsos, 2001; Heene et al., 2011).

Given that the empirical foundation for ACM might seldom be given it is then reasonable to apply more flexible measurement models such as the Rasch model (Rasch, 1981) which some authors regard as a *probabilistic* formulation of ACM (Perline et al., 1979) and also leading to interval-level measurement. Kyngdon (2008b), however, argues that there is no basis for this claim by showing that parameters of IRT and Rasch models are only invariant against positive monotone transformations. Thus, if both the Rasch model and the more general three-parameter logistic model fit a data set, only the *order* upon the person ability estimates produced by these models remains invariant. Hence, as only order is preserved under positive monotone transformation (Narens, 1981), the fit of an IRT or a Rasch model, respectively, may in fact not be indicative of quantity, but of order.

Moreover, justification for using the Rasch model relates frequently to the argument that random error forms a fundamental that is, non-ignorable feature of every psychological response process and must therefore be included in any model formulation (cf. Borsboom and Scholten, 2008; Fisher, 2011). Since the Rasch model as a probabilistic model accounts for random error it seems to be the panacea of the measurement problems in psychology. However, the magic of obtaining an interval-scale for items and examinees comes with a price because the Rasch model's status as a quantitative theory is derived exclusively through the error term as Michell (2008) pointed out. With the Rasch model, if the error was eliminated, the slope of the item response curves would become infinite, resulting in step-functions of the Guttman model and the “measurements” of the Rasch model reduce only to mere order. But eliminating error must by definition lead to better measurement, not the impossibility of measurement. Nevertheless, Sijtsma (2012) has recently argued that this reasoning is incorrect:

The Guttman model divides the latent variable scale into disjoint and exhaustive intervals in which differences Θ − δ_*j*_ do not affect response probabilities. The Rasch model assumes these differences to have a monotone relationship to response probabilities. From the viewpoint of IRT, the Guttman model ignores the information contained in the intervals, thus paying the price of a lower measurement level. (p. 14)

I do not see why this line of argumentation refutes Michell's (2008) “Rasch paradox”. Sijtsma's reasoning *presupposes* that the latent trait is continuous. Furthermore, we can only ignore information “… contained in the intervals” when there already is interval-level information, but this is not at all self-evident but simply an assumption of IRT.

This uncomfortable situation that psychometric models cannot work without “error,” has lead in my opinion, to great statistical hand wringing and argumentative acrobatics to avoid falsification of the quantitaty assumption. This line of argumentation is often linked to the demonstration of correspondences between psychology and physics.

For instance, Fisher (2011) claims that the probabilistic nature of the Rasch model reflects the physical phenomenon of stochastic resonance (SR) within a biological system. Simply put, SR states that an output signal-to-noise ratio of a nonlinear threshold system is improved by moderate values of input noise intensity (cf. McNamara and Wiesenfeld, 1989). The weak and normally undetectable signal becomes then detectable due to resonance between the signal and the added stochastic noise because the added noise will occasionally lead to an exceeding of a threshold value of the periodic force (see Gammaitoni et al., 1998, for illustrative examples). A plethora of physical, biological and neurophysiological systems, as well as some phenomena from linguistics and visual perception can be described by SR which has been indirectly shown by applying both the signal and the noise externally to receptors and neurons or by data simulations (cf. Simonotto et al., 1997; Gammaitoni et al., 1998; Moskowitz and Dickinson, 2002).

Although it is intriguing to regard SR as a valid justification for probabilistic item response models in order to capture randomness, such an extrapolation is far-fetched because it is not at all self-evident why and how such micro-level phenomena can be extrapolated to the macro-level of item responses. Moreover, because present results on SR in biological systems bear on indirect evidence, the general applicability of SR to such systems is far from being clear as noted by McDonnell and Abbott (2009):

Adding noise to external stimuli cannot prove that neurons or brain function depend on consistently available internal sources of randomness, i.e., on endogenous neural noise. The challenge is to devise an experiment that can remove naturally occurring healthy variability and demonstrate that function is impaired solely due to that removal. (p. 6)

It appears that borrowing examples from the natural sciences and relating them to the (error) structure of probabilistic item response models might be a persuading analogy but is not a convincing justification for the probabilistic nature of item response models. Explicit cognitive theories of the test item response process are needed, but psychometrics is profoundly lacking in such theories (Kyngdon, 2011). Furthermore, no experimental evidence currently exists which shows why and how such system-inherent error might occur in the item response process.

Finally, I just wonder why psychometricians have yet ignored the success ACM has within theories of utility and decision making in psychology (“prospect theory”; Kahneman and Tversky, 1979) in which ACM served as a formal proof. While it is true that human choice behavior did not strictly follow the requirements of ACM and research has discovered paradoxes of human choice behavior (Birnbaum, 2008), it is also clear that these observations have led to falsifications of old theories of choice behavior and the development of new ones that account for persistent violations of coalescing and first order stochastic dominance (e.g., Birnbaum, 2008; Luce et al., 2008). Frankly speaking, I have very rarely seen such an attitude within mainstream psychometrics be it IRT/Rasch or SEM where items are omitted from tests, powerless but flattering item-fit statistics are commonly used (Karabatsos, 2001), and correlated error terms are specified (Cole et al., 2007) to get a reasonable model-fit and to construct support for one's own the theory despite doubtful consequences (cf. Bones, 2012; Ferguson and Heene, 2012).

## Conclusion

Altogether, it is possible that human cognitive abilities and personality traits simply are not quantitative. ACM might be in fact too severe for practical testing purposes. However, psychometricians continue to argue that cognitive abilities are quantitative and measurable “latent traits” (Markus and Borsboom, 2012). If this argument is correct, then once item response error is controlled, test score response data should be consistent with the cancellation axioms of ACM. Thus, more direct experimentation is needed instead of more sophisticated IRT models.

It is still unclear and an unsolved problem what SEM and IRT models, notably the Rasch model, add to the clarification of the quantity problem in psychology. It is furthermore unclear what insights into *empirical phenomena* it provides as even attempts to explain the error structure seem to be premature. It is mostly forgotten that Rasch himself did not derive his model from empirical observations but “… within [Rasch's] own mathematical playground—with no relation to any actual item analysis problem!” (Rasch, 1979). It is not necessarily wrong to develop mathematical models *independently* from empirical observations. But, it is also not at all self-evident that empirical insights will result from such models, be it an IRT, SEM, or ACM. However, by avoiding tests of the assumption of a quantitative structure of psychological attributes, psychologists have yet failed to make progress on the basis of the fundamental scientific principle of falsification and in regard to their most fundamental assumptions of quantitative psychological attributes.
